# Successful laparoscopic removal of ovarian borderline cystadenoma in patient with multiorgan hemangioma and ventriculoperitoneal shunt: a case report and literature review

**DOI:** 10.1093/jscr/rjab380

**Published:** 2021-11-11

**Authors:** Chenyi Zhang, Xinhui Jing

**Affiliations:** Department of Obstetrics and Gynecology, Qilu Hospital of Shandong University, Jinan, Shandong, P.R. China; Department of Obstetrics and Gynecology, The Third Hospital of Jilin University, Changchun, Jilin, P.R. China

## Abstract

As medical diagnosis and treatment level improved, patients with ventriculoperitoneal shunt (VPS) live longer and may develop conditions that need laparoscopic surgery. The safety of laparoscopy in patients with VPS continues to be challenged due to pneumoperitoneum. Here, we report a patient with medical history of VPS and hemangioma, diagnosed with ovarian borderline mucinous cystadenoma, received laparoscopic surgery in supine position and 10 mmHg pneumoperitoneum pressure, in which no clamping or externalizing catheter, no perioperative or postoperative complications. We also present a literature review and discuss the precautions needing considering during laparoscopy. For patients with VPS, laparoscopic surgery can be recognized as a potentially safe and feasible procedure.

## INTRODUCTION

Ventriculoperitoneal shunt (VPS) is a medical device relieving pressure on the brain caused by fluid accumulation, some of which have valves that can be adjusted to modify the rate of fluid flow. It is widely applied to treat hydrocephalus but controversial for the safety of laparoscopic surgery. Some believe pneumoperitoneum can give rise to potential problems, such as elevated intracranial pressure (ICP), pneumocephalus and retrograde infection [1, 2]. Others consider it safe [3–5]. Here, we report a case about a VPS patient with complicated multiorgan hemangioma successfully underwent laparoscopic surgery.

## CASE REPORT

**Figure 1:**
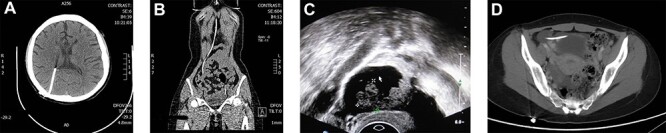
(**A** and **B**) CT showed the shunt from lateral ventricle to abdominal cavity; (**C**) Ultrasound of gynecology showed a mass measuring about 3.5 × 2.3 cm in the right adnexa, which was mainly cystic, with solid irregular protrusions ranging from 2.7 × 1.6 cm inside, in which blood flow signals were explored; (**D**) Pelvic CT scan suggested drainage catheter was located in the front of the uterus.

A 25-year-old unmarried female came to our hospital with a complaint of gradual enlarging mass in right adnexa about 1 year without pain. The patient had medical history of receiving VPS 16 years ago due to hydrocephalus (Fig. 1a and b). Laboratory results were as follows: cytosine arabinoside (CA) 19–9: 49.34 U/ml (0–35) and CA 724: 19.6 U/ml (0–6.9). Hematopoiesis and clotting activity, as well as functions of the liver and kidneys were all normal. The results of ultrasound of gynecology and pelvic CT scan are shown (Fig. 1c and d). Abdominal ultrasound indicated multiple angiomas were observed in the liver and spleen.

**Figure 2:**
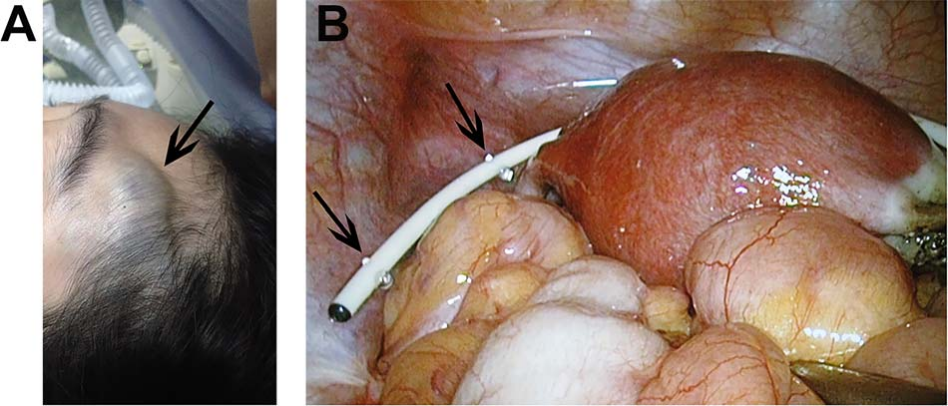
(**A**) Swelling forehead hemangioma in Trendelenburg position; (**B**) The end of the catheter was exposed and clear cerebrospinal fluid was dripped from the catheter continuously.

During the surgery, the patient was placed from Trendelenburg position to supine position because the patient’s forehead hemangioma was swelling sharply in Trendelenburg (Fig. 2a). The current procedure was performed using standard 4 port technique. The pneumoperitoneum was maintained at 10 mmHg. Throughout the operation, the end of the catheter was exposed, clear cerebrospinal fluid was dripped from the catheter continuously (Fig. 2b). After excising the right adnexa, rapid pathology revealed right ovary borderline mucinous cystadenoma. The patient was discharged on the third day after surgery. In the recent follow-up, 5-year after surgery, the patient was still in good condition with no complications or relapse.

## DISCUSSION

In the laparoscopic surgery, the main differences between VPS patients and non-VPS patients are the history of craniocerebral disease and the exist of a drainage catheter. Concerns regarding the increase of ICP and safety of abdominal insufflation in VPS patients were raised. In 1994, Josephs firstly used pigs to prove that pneumoperitoneum could increase ICP [6]. In 1997, Uzzo proposed that elevated intra-abdominal pressure (IAP) would lead to an increase in ICP [7]. In 2000, Jackman monitored ICP of 18 VPS patients during laparoscopic surgery. The results showed that IAP could cause a transient increase in ICP, but no sustained postoperative elevation and nervous injury [3]. In 2014, a study illustrated that ICP ranged from 0 to 18 cmH2O with desufflation and from 8 to 25 cmH2O with 15 mmHg CO_2_ insufflation [8]. For patients with head injury, decreasing ICP to <20 mmH2O and keeping cerebral perfusion pressure at >50 mmHg is necessary according to Brain Trauma Foundation [9, 10]. In our case, we maintained the pneumoperitoneum at 10 mmHg and did not affect the operation. For conclusion, though most of ICP elevations may not be clinically significant, lower pneumoperitoneum should be chosen as much as possible after meeting the basic surgical requirements.

During laparoscopic surgery, it is still controversial whether the countercurrent flow of gas and liquid occurred due to the potential pressure between ICP and IAP. It has been reported that a VPS patient developed pneumocephalus after laparoscopic surgery [2]. The author analyzed the reason was that the drainage catheter has existed in the patient for over 20 years and was ineffective. Nevertheless, Collure *et al.* showed that the one-way valve of the drainage catheter can withstand pressure up to 300 mmHg. It is unlikely that pneumocephalus will be produced by the CO2 insufflation at pressure of 10–15 mmHg during laparoscopy [11]. Due to the pressure difference, in addition to the protection of the drainage valve, the possibility of CO2 counterflow is little. In another study, a simplified reflux experiment was performed using a clinical pneumoperitoneum device. No reflux occurred when the catheter was filled with salt water. This fact also indicates that no reflux would occur during operation due to flow of CSF of the shunt system [12]. However, for the VPS patients whose catheters lack of one-way valves, or have been placed for a long time and the valves do not work anymore, it should be paid more attention to the management of catheters during the laparoscopic surgery, such as clamping the catheters, or removing the catheters from the abdominal cavity before surgery. Besides, Laparoscopic surgery should be postponed for patients who have recently completed VPS surgery until the tract has fibrosed and sealed, although the exact timing for this has not been determined. Two failed laparoscopic surgeries were reported, which were carried out at 5 and 10 days after VPS surgery, respectively [1, 13].

As the only connection between ventricular and abdominal cavity, catheter’s unobstructed and sterility are crucial. Current reports have brought many new insights into the management of the drainage catheter, including the distal end of the catheter [14, 15], placing the distal end of the catheter into the Endopouch bag [16], as well as introduction of Lap Disc to optimize the process of externalizing shunt [17]. Besides Baskin JJ reported a case with no special handle of the catheter and acute obstruction of the catheter happened because of impaction of soft tissue within the distal catheter as a consequence of peritoneal insufflation [1]. So, is it necessary to handle the catheter during laparoscopic surgery? When externalization the catheter, the disadvantage is possibility of infection. Actually, most of the reports we searched had no manipulation of the catheters during laparotomy and laparoscopic surgery as well as robotic surgery no matter in adults or children who are susceptible to infection (Table 1). A study from Stanford University found it was at very low risk for catheter malfunction or infection in VPS patients receiving clean or clean-contaminated surgeries and externalization of the shunt was unnecessary [18].

**Table 1:** Cases of laparoscopic surgery in patients with VPS reported in literature

Authors	Year	Number of patients	Age (years)	Surgical indication	VPS indications	Pneumoperitoneum pressure (mmHg)	Catheter manipulation	Postoperative complications	Special measures during laparoscopy
Schwed, D. A. *et al.*	1992	1	73	Cholecystitis	Hydrocephalus	10–15	None	Subcutaneous emphysema	Recent VPS surgery may be a relative contraindication to laparoscopic surgery.
Collure, D. W *et al.*	1995	3	74	Cholecystitis with cholelithiasis	Hydrocephalus	10–15	None	None	None
			75	Acute cholecystitis	Hydrocephalus	10–15	None	None	None
			39	Acute cholecystitis and cholelithiasis	Hydrocephalus	10–15	None	None	None
Tobias, D. H. *et al.*	1996	1	64	Pelvic mass	Hydrocephalus	15	None	None	Exploratory laparotomy in the surgery due to multiple adhesions from previous surgery
Baskin, J. J. *et al.*	1998	1	52	Jejunostomy	Hydrocephalus	15	None	Distal shunt obstruction	Gentle irrigation cleared an acute distal catheter obstruction
Walker, David H. *et al.*	2000	10	16 months–16 years	Fundoplication(6)cholecystectomy(2), malrotation(1), diagnostic laparoscopy(1).	Not referred	10–15	None	None	None
Jackman, S. V. *et al.*	2000	18	13.2	Not referred	Not referred	12–20	None	None	None
Nawashiro, H. *et al.*	2002	1	61	Pancreatic carcinoma	Hydrocephalus	Not referred	None	Seeding of pancreatic carcinoma along shunt catheter	None
Al-Mufarrej, F. *et al.*	2005	1	34	Acute cholecystitis	Arnold-Chiari malformation, epilepsy, syringomyelia, and posterior fossa decompression	13	Clamp	None	None
Eralp, Y. *et al.*	2008	1	36	Metastatic carcinoma	Non-communicating hydrocephalus	Not referred	None	Leptomeningeal dissemination of ovarian carcinoma	Intraperitoneal chemotherapy may be an alternative treatment modality which would decrease the cellular migration
Mislow, J. M. *et al.*	2009	1	21	Ovarian teratoma	Hydrocephalus	Not referred	None	None	Misdiagnose cystic ovarian neoplasm as cerebrospinal fluid pseudocyst
Hammill, C. W. *et al.*	2010	1	71	Cholelithiasis	Hemorrhagic cerebrovascular accident	Not referred	None	None	None
Raskin, J. *et al.*	2010	1	24	Endometriosis	Cerebral palsy, myelomeningocele and hydrocephalus	50	None	Pneumocephalus	None
Orbuch, I. K *et al.*	2010	1	42	Endometriosis	Chiari IImalformation	Not referred	Externalize shunt through LapDisc	None	None
Smiljanic, A. *et al.*	2011	1	71	Cholelithiasis	Hemorrhage cerebrovascular accident	Not referred	None	None	None
Ghomi, A. *et al.*	2011	1	21	Symptomatic pelvic organ prolapse	Spida bifida	5–15	None	None	Intraperitoneal pressure decreased every 30 min
Damrah, O. *et al.*	2011	1	64	Acute cholecystitis	Subarachnoid hemorrhage(SAH)	12–15	None	None	None
Marchetti, P. *et al.*	2011	4	8	Hydronephrosis	Myelomeningocele	12	Distal end of the shunt was placed into an Endopouch bag	None	None
			7	Hydronephrosis	Myelomeningocele				
			14	Urinary retention	Arnold-Chiari malformation				
			14	Urinary-tract infection	Myelomeningocele				
Staikou, C. *et al.*	2012	1	40	Adrenal gland adenoma	Hydrocephalus	10–14		None	Transcranial Doppler (TCD)
Magnani, C. *et al.*	2012	1	44	Cholelithiasis	Hydrocephalus	7–8	None	None	None
Torigoe, Takayuki *et al.*	2013	1	59	Cecal cancer	Hydrocephalus	Not referred	Clamp	None	None
Mungasuvalli, N. C. *et al.*	2014	1	24	Von Hippel Lindau (VHL) syndrome	Haemangioblastomas	Not referred	None	None	Confirm the patency of the VP shunt by demonstrating the drainage of the CSF into the peritoneal cavity
Cobianchi, Lorenzo. *et al.*	2014	1	41	Gallbladder polyps	Hydrocephalus	12	None	None	None
Albarrak, Abdullah A. *et al.*	2015	1	41	Acute calcular cholecystitis	Pseudotumor cerebri	12	None	None	Supine position
Yoshihara, Terukazu. *et al.*	2017	2	56	Gallbladder stone	Hydrocephalus	Not referred	Clamp	None	None
			73	Gallbladder stone	Hydrocephalus	Not referred	Clamp	None	None
Fuad, Shalabi *et al.*	2018	2	71	Colon cancer	Hydrocephalus	Not referred	None	None	None
			78	Colon cancer	Not referred	Not referred	None	None	None
Ishikawa, T. *et al.*	2018	1	77	Rectal cancer	Hydrocephalus	10	None	None	In a 15° head-down tilt
Monsellato, I.	2019	1	74	Colon tumor	Hydrocephalus	8–10	None	None	Supine position with a slight Trendelenburg (5 degrees) and a 5-degrees left tilt
Rosenfeld, E. H. *et al.*	2019	25	0.9–5.8	Gastrostomy	Not referred	Not referred	None	None	None

Furthermore, surgical position is also very important. A recent study shows that Trendelenburg position will aggravate ICP elevation during gynecological laparoscopic surgery [19]. We used supine position during the operation and found it fulfilled the requirement of surgical field exposure and was crucial to patients of multiorgan hemangioma.

In conclusion, based on our clinical experience and published studies, laparoscopic surgery is safe and effective for VPS patients with adequate preoperative evaluation and intraoperative manipulation.
